# Generation of reactive oxygen species in 1-methyl-4-phenylpyridinium (MPP+) treated dopaminergic neurons occurs as an NADPH oxidase-dependent two-wave cascade

**DOI:** 10.1186/1742-2094-8-129

**Published:** 2011-10-05

**Authors:** W Michael Zawada, Gregg P Banninger, Jennifer Thornton, Beth Marriott, David Cantu, Angela L Rachubinski, Mita Das, W Sue T Griffin, Susan M Jones

**Affiliations:** 1 Donald W. Reynolds Department of Geriatrics, University of Arkansas for Medical Sciences, Little Rock, AR 72205, USA; 2 Department of Pharmacology and Toxicology, University of Arkansas for Medical Sciences, Little Rock, AR 72205, USA; 3 Neurotrauma Research, Swedish Medical Center, Englewood, CO 80113, USA

## Abstract

**Background:**

Reactive oxygen species (ROS), superoxide and hydrogen peroxide (H_2_O_2_), are necessary for appropriate responses to immune challenges. In the brain, excess superoxide production predicts neuronal cell loss, suggesting that Parkinson's disease (PD) with its wholesale death of dopaminergic neurons in substantia nigra pars compacta (nigra) may be a case in point. Although microglial NADPH oxidase-produced superoxide contributes to dopaminergic neuron death in an MPTP mouse model of PD, this is secondary to an initial die off of such neurons, suggesting that the initial MPTP-induced death of neurons may be via activation of NADPH oxidase in neurons themselves, thus providing an early therapeutic target.

**Methods:**

NADPH oxidase subunits were visualized in adult mouse nigra neurons and in N27 rat dopaminergic cells by immunofluorescence. NADPH oxidase subunits in N27 cell cultures were detected by immunoblots and RT-PCR. Superoxide was measured by flow cytometric detection of H_2_O_2_-induced carboxy-H_2_-DCFDA fluorescence. Cells were treated with MPP+ (MPTP metabolite) following siRNA silencing of the Nox2-stabilizing subunit p22^phox^, or simultaneously with NADPH oxidase pharmacological inhibitors or with losartan to antagonize angiotensin II type 1 receptor-induced NADPH oxidase activation.

**Results:**

Nigral dopaminergic neurons *in situ* expressed three subunits necessary for NADPH oxidase activation, and these as well as several other NADPH oxidase subunits and their encoding mRNAs were detected in unstimulated N27 cells. Overnight MPP+ treatment of N27 cells induced Nox2 protein and superoxide generation, which was counteracted by NADPH oxidase inhibitors, by siRNA silencing of p22^phox^, or losartan. A two-wave ROS cascade was identified: 1) as a first wave, mitochondrial H_2_O_2 _production was first noted at three hours of MPP+ treatment; and 2) as a second wave, H_2_O_2 _levels were further increased by 24 hours. This second wave was eliminated by pharmacological inhibitors and a blocker of protein synthesis.

**Conclusions:**

A two-wave cascade of ROS production is active in nigral dopaminergic neurons in response to neurotoxicity-induced superoxide. Our findings allow us to conclude that superoxide generated by NADPH oxidase present in nigral neurons contributes to the loss of such neurons in PD. Losartan suppression of nigral-cell superoxide production suggests that angiotensin receptor blockers have potential as PD preventatives.

## Background

Reactive oxygen species (ROS) contribute to cellular signaling, affecting most aspects of cellular function including gene expression, proliferation, differentiation, and migration [1,2]. Under normal physiological conditions, such effects may be beneficial, but an excess of ROS can negatively affect cell function and survival by damaging cellular macromolecules: lipids, nucleic acids, and proteins [3-5]. For example in response to bacterial infection in the brain, a bactericidal oxidative burst is generated by activated microglia [6] and the superoxide produced in this burst results in the oxidative stress, which unabated results in progressive neuronal distresses such as those in PD [7-9]. The oxidative burst induced in activated phagocytes such as neutrophils [10] and microglia [11] comes from superoxide-generating NADPH oxidase.

The NADPH oxidase enzyme consists of several subunits, two of which are permanently membrane bound: the catalytic Nox2 (gp91^phox^) subunit and the Nox2-stabilizing p22^phox ^subunit. Nox2 has six membrane-spanning domains, two hemes, and a NADPH binding site [12]. Nox2 interaction with p22^phox ^forms a cytochrome b558 complex, which is necessary for NADPH oxidase activity for production of superoxide through recruitment of a small GTPase Rac2, and of p47^phox ^and p67^phox ^to the plasma membrane [13]. Formation of the NADPH oxidase complex may involve alternative isoforms of the component subunits [14]. The current database of the human genome contains seven members of the NADPH oxidase family. The members include Nox1-5, together with two dual oxidases (Duox1 and 2) that contain both NADPH oxidase and peroxidase-like domains [14,15]; the tissue distribution of these seven family members varies significantly [14]. The gene encoding Nox5 is not present in rodents [16]. Although several pharmacological inhibitors of NADPH oxidase exist [17,18], their specificity, efficacy, and safety differ widely. An alternative and potentially sounder approach to suppression of NADPH oxidase-generated superoxide utilizes angiotensin II type 1 (AT1) receptor blockers, exemplified by the original compound in this class, losartan [19,20]. This is possible because generation of superoxide from NADPH oxidase is promoted by angiotensin II binding to the AT1 receptor, leading to induction of protein kinase C-induced Nox2 signaling [19]. Antagonists of the AT1 receptor such as candesartan and losartan suppress angiotensin II-induced increases in superoxide production and Nox2 expression [21].

Postmortem analysis of the midbrain of PD patients has provided evidence of microglial activation in this pathogenic process [22-26]. This activation of microglia, the macrophage-like, resident immune cells of the brain, and ROS production has been associated with the neurodegeneration characteristic of PD [27]. In response to brain injury and immunological challenges, microglia become readily activated and produce a wide array of cytokines and cytotoxic factors, including ROS as well as TNF-α, eicosanoids, IL-1β, and nitric oxide [28-30]. In one model of dopaminergic degeneration, activation of microglia by the inflammatory factor lipopolysaccharide is rapid and is followed by a delayed, progressive, and selective destruction of nigral dopamine neurons both *in vitro *and *in vivo *[31]. Microglial activation significantly enhances MPP+ (1-methyl-4-phenylpyridinium, a metabolite of MPTP, 1-methyl-4-phenyl-1,2,3,6-tetrahydropyridine) damage to dopaminergic neurons in a primary neuron-glia cell culture model of dopaminergic cell death [7]. However, this occurs not by direct activation of microglia by MPP+, but rather as a result of microglial stimulation by factors released from an initial die off of dopaminergic neurons. As a result of this sequential neuronal-glial interaction, the primary damage to even a few dopaminergic neurons leads to extensive microglia-enhanced neurodegeneration [7]. Importantly, these findings suggest that ROS responses in dopaminergic neurons, themselves, are a necessary initial step in a cascade that leads to the flagrant neuronal cell loss in response to MPP+ treatment. A link between microglia and NADPH oxidase as mediators of neurotoxicity in experimental models of PD is further supported by findings of a reduction in the loss of dopamine neurons upon exposure to MPP+ in mesencephalic neuron/glia cultures derived from Nox2-deficient mice. In addition, these mice are partly resistant to MPTP treatment [7,8]. The binding of MPP+ to the mitochondrial electron transport chain complex I results in decreased production of ATP, elevation in superoxide generation, and subsequently cell death.

Babier *et al*., suggests that NADPH oxidase-induced ROS initially developed as a universal signaling mechanism in all cell types and evolved in macrophages as a means of cellular defense [32,33]. In the CNS, cerebral cortical neurons [34] as well as hippocampal pyramidal neurons [35], cerebellar Purkinje cells [36,37], central autonomic neurons of the intermediate dorsomedial nucleus of the solitary tract [38], and neonatal sympathetic neurons express various subunits of the non-phagocytic NADPH oxidase [39]. While little is known regarding the functions of these subunits in these neuronal cells, two studies point to potential roles in memory formation in the hippocampus [40] and maintenance of growth cone dynamics by F-actin in a giant neuron of a sea snail, *Aplysia *[41]. The function(s) of the NADPH oxidase Nox2 subunit identified in hippocampal and cortical astrocytes [42] remains undefined. Although microglial NADPH oxidase participates in dopaminergic neurotoxicity [43], whether it also exists in dopamine neurons and contributes to the ROS production in the midbrain has not been explored [8].

In this study, we found NADPH oxidase subunits in tyrosine hydroxylase (TH)-immunoreactive neurons of the adult mouse nigra. In addition, expression of NADPH oxidase in a rat nigral dopaminergic cell line, N27, allowed us to investigate the potential role of NADPH oxidase in generation of the MPP+ induced ROS. We found that: *i*) N27 cells express all the components of NADPH oxidase that are required for its activation; *ii*) that treatment of these cells with NADPH oxidase inhibitors, or with an angiotensin II type 1 receptor blocker leads to an attenuation of MPP+ induced generation of hydrogen peroxide (H_2_O_2_, a product of superoxide dismutation); *iii*) MPP+ treatment induced a biphasic (two-wave) generation of H_2_O_2_; and *iv*) NADPH oxidase inhibitors blocked selectively only the second wave of H_2_O_2 _production. These findings support our hypothesis that neuronal NADPH oxidase plays an important role in neuronal stress responses, which contribute to vulnerability of dopaminergic neurons in PD.

## Materials and methods

### Animals and cell culture

Animal protocols and use were in strict accordance with the NIH *Guide for the Care and Use of Laboratory Animals *and were approved by the Institutional Animal Care and Use Committee at the University of Colorado Denver. We used female C57BL/6J mice from Jackson Laboratories (6 weeks old; 15-18 g) for studies of NADPH oxidase subunit expression in the substantia nigra. Mice were housed individually on a 12 h light/dark cycle with food and water available *ad libitum*.

For all cell culture experiments, we used the N27 dopaminergic cell line derived from rat ventral mesencephalon at gestational day 12. This cell line is often used to model dopaminergic neurons because it expresses the dopaminergic markers TH and plasma membrane dopamine transporter, and produces dopamine [44]. N27 cells were grown in RPMI medium (CellGro) containing 10% fetal bovine serum (CellGro), penicillin-streptomycin, L-glutamine, and 1 μM angiotensin II (Sigma) in a 37°C incubator and 5% CO_2_. Cell counts were conducted after trypsinizing N27 cells and counting cells under a hemocytometer. To generate ROS in N27 cell cultures, we treated N27 cells for upto 24 hours with a range of concentrations of MPP+, a metabolite of MPTP. The mechanism by which MPTP exerts toxicity *in vivo *requires its conversion in astrocytes via monoamine oxidase B to MPP+ (reviewed in [45]). Since the N27 cultures lack astrocytes to perform this conversion, we have treated N27 cells with MPP+ itself. Specific dopaminergic neurotoxicity caused by MPP+ depends on the selective uptake of MPP+ via the dopamine transporter into the cytosol where it concentrates inside the mitochondria.

### Reagents and antibodies

MPP+, cyclohexamide (c-hex), apocynin, phenylarsine oxide (PAO) and losartan potassium were obtained from Sigma. Anti-p47^phox ^and anti-Nox2 antibodies were from Upstate Biotechnology; anti-p22^phox ^and anti-p67^phox ^antibodies were from Santa Cruz Biotechnology; and anti-TH from Pel-Freez Biologicals. Alkaline phosphatase conjugated anti-rabbit antibody was purchased from Chemicon, anti-goat antibody from Jackson Immunoresearch Laboratories, Lumi-Phos™ WB from Pierce, propidium iodide from Becton Dickinson, 5-(and-6)-carboxy-2',7'-dichlorodihydrofluorescein diacetate (carboxy-H_2_-DCFDA) substrate dye from Molecular Probes, and Hoechst 33258, and secondary antibodies (Alexa 488 and 568) from Invitrogen.

### Immunofluorescent staining

Six-week-old female C57BL/6J mice were deeply anesthetized and transcardially perfused with saline followed by 4% paraformaldehyde. The brains were cryoprotected in 30% sucrose for 2 days before the frozen midbrain region containing nigra was sectioned coronally into 40 μm-thick sections. Floating sections were rinsed, blocked for 20 minutes in 10% normal goat serum in Tris-buffered saline (TBS) containing 1% BSA and 0.1% Triton X-100, rinsed again, and incubated overnight at room temperature with the following primary antibodies: mouse monoclonal anti-Nox2 (1:750), mouse monoclonal anti-p47^phox ^(1:200), mouse monoclonal anti-p67^phox ^(1:750), or rabbit polyclonal anti-TH (1:500). The secondary antibodies were anti-rabbit Alexa 488 (green) and anti-mouse Alexa 568 (red). Fluorescence was imaged in the sections using a Zeiss LSM 510 confocal microscope.

For the immunofluorescent staining of N27 cells grown in 96-well plates, the cultures were rinsed with PBS, fixed in 4% paraformaldehyde for 1 hour, blocked for 20 minutes with the aforementioned goat serum preparation and incubated with primary antibodies to Nox2, p22^phox^, and p47^phox ^followed by secondary antibodies as described for the brain sections above. Hoechst 33258 was used to visualize cell nuclei. Images of immunofluorescent detection of antigens and nuclei in N27 cells were acquired using a SPOT camera attached to an inverted Nikon Eclipse TS100 epi-fluorescence microscope.

### ROS measurements

Flow cytometry with carboxy-H_2_-DCFDA detection identified intracellular H_2_O_2_, which was used as a surrogate marker for superoxide generation. Oxidation of this non-fluorescent substrate generates a green fluorescent product. Because the cell membranes are permeable to the esterified form of carboxy-H_2_-DCFDA, cells uptake it freely. The dye becomes trapped in the cells as a result of deacetylation by intracellular esterases and thus becomes available to oxidation by intracellular H_2_O_2_. Since superoxide is relatively short lived because it is rapidly dismutated to H_2_O_2_, intracellular H_2_O_2 _levels are therefore a more reliable indicator of intracellular ROS burden. For the flow cytometry assay, cells were trypsinized using 0.25% trypsin-EDTA solution (CellGro) and resuspended in growth medium. Cells (5.0 × 10^6^) were delivered into 5 mL polystyrene tubes, pelleted, and then incubated for 25 minutes at 37°C with carboxy-H_2_-DCFDA mixed isomers reagent diluted to 2 μM in PBS supplemented with 0.5% FBS (PBS-FBS). Cells were then pelleted again and incubated with growth media for 10 minutes at 37°C and washed twice with PBS-FBS. In the final step, cells were pelleted, resuspended in propidium iodide solution for 10 minutes, and carboxy-H_2_-DCFDA fluorescence emitted by 10,000 live cells was quantified using BD FACScan flow cytometer.

### Reverse Transcription PCR and Western blotting

Total RNA was collected from N27 cells following the Trizol protocol (Invitrogen). Messenger RNA was reverse transcribed using random primers to cDNA with Superscript II (Invitrogen). PCR was performed using NADPH oxidase subunit gene specific primers that yielded PCR products ranging between 400 and 500 bp. The following primer sets were designed based on the following accession numbers: rat Nox1 [GenBank:NM_053683] forward: 5'-AGCCATTGGATCACAACCTC-3'*/ *reverse: 5'-TGAGGCTCCTGCAACTCCT-3'; rat Nox2 [GenBank:NM_023965] forward: 5'-GTGGAGTGGTGTGTGAATGC-3'/reverse: 5'-AGGATGAGTGACCACCTTGG-3'; rat Nox3 [GenBank:NM_001004216] forward: 5'-TCTGTAGCATGCCGAGACTG-3'/reverse: 5'- AATGAACGCCCCTAGGATCT-3'; rat Nox4 [GenBank:NM_053524] forward: 5'-TGTCTGCTTGTTTGGCTGTC-3'/reverse: 5'-AGCAGCAGCAGCATGTAGAA-3'; rat p22^phox ^[GenBank:AJ295951] forward: 5'-TTGTTGCAGGAGTGCTCATC-3'/reverse: 5'-CGACCTCATCTGTCACTGGA-3'; rat p40^phox ^[GenBank:NM_001127304] forward: 5'-ATGGAAGCTCCAAGAGCAGA-3'/reverse: 5'- AATTGTCCTTCTGGGTGACG-3'; rat p47^phox ^[GenBank:NM_053734] forward: 5'-AGCTCCCAGGTGGTATGATG-3'/reverse: 5'-TGTCAAGGGGCTCCAAAT-3'; and rat p67^phox ^[GenBank:NM_001100984] forward: 5'-TCATGCATGCCAAGAAAGAG-3'/reverse: 5'-CCCTTCTGTCCGTTGAACAT-3'. PCR products were separated by electrophoresis on 1% agarose gels and visualized with ethidium bromide. Specificity of each primer set was confirmed by amplification of a single band of expected size.

For Western immunoblots, whole cell lysates were made in cell lysis buffer (Promega) supplemented with 1% SDS, sonicated and separated on precast 4-12% SDS-PAGE gels (Invitrogen). Proteins were then transferred to PVDF membranes and blocked with 5% milk in TBS with 1% Tween-20 (TBST). The membranes were then incubated overnight at 4°C with the following primary antibodies: anti-p47^phox^, anti-Nox2 (both 1:1000), and anti-p22^phox ^and anti-p67^phox ^(1:500 and 1:200, respectively). Membranes were then rinsed, incubated in alkaline phosphatase-conjugated secondary antibodies (1:10,000), and a chemiluminescent signal was detected using Lumi-Phos™ WB.

### siRNA transfection

N27 cells were plated at 35,000 cells/cm^2 ^in antibiotic-free RPMI growth medium containing 5% fetal bovine serum. SiRNA duplexes (siGenome On-Target Smartpool duplex 9 CYBA/p22^phox ^and On-TARGETPlus siControl Non-Target Pool; Dharmacon, Inc) were resuspended in siRNA universal buffer (Dharmacon, Inc) at 20 μM and stored in aliquots at -20°C. Cells (50-60% confluent) were transfected twice at 24-hour intervals with 100 nM siRNA and 2 μl Lipofectamine 2000 per ml of Opti-MEM according to the manufacturer's instructions (Invitrogen). After overnight transfection, media were replaced with fresh media for 4-6 hours before second overnight transfection. After the second transfection, cultures were treated with MPP+ for 18 hours and ROS measured as described above. Knockdown of p22^phox ^expression was verified using RT-PCR as described above.

### Statistics

All data are expressed as means +/- SEM of three independent experiments. One-way ANOVA was used for statistical comparisons of multiple groups followed by a Student-Newman-Keuls post-hoc test. Mean values were considered statistically different when p < 0.05.

## Results

### NADPH oxidase subunits are expressed in both dopaminergic neurons in the mouse substantia nigra pars compacta and in the rat dopaminergic cell line N27

**Figure 1:**
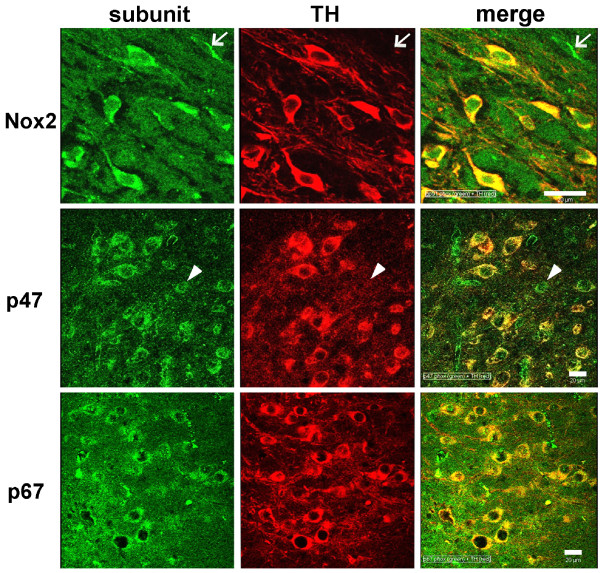
**Dopamine neurons in the adult female C57BL/6J mouse substantia nigra express Nox2, p47^phox ^and p67^phox ^subunits of the NADPH oxidase**. Immunofluorescent histochemical staining revealed the presence of Nox2, p47^**phox **^and p67^**phox **^(green) in the neurons positive for TH (red). Merged images indicate co-labeling (yellow). Occasionally cells positive for a subunit were not dopaminergic neurons (TH^-^cells). To illustrate such an example, arrows in the top row point to a Nox2^+^/TH^- ^cell and arrowheads in the middle row indicate a cell that is p47^phox+^/TH^-^. Scale bars equal 20 μm.

NADPH oxidase is present in microglia [11], astrocytes [46], and in certain types of neurons in hippocampus and cortex [46,47]. Here, in adult mice, we show that dopaminergic TH immunoreactive neurons in substantia nigra coexpress three of the NADPH oxidase subunits, *viz*., Nox2, the catalytic subunit responsible for superoxide generation, as well as the two subunits, p47^phox ^and p67^phox^, necessary for Nox2 activation (Figure 1). The expression was predominantly cytoplasmic, no nuclear localization was observed, and negative control staining by omitting the primary antibody did not produce detectable immunoreactivity (data not shown). While all TH-immunoreactive neurons expressed these subunits, occasionally cells lacking TH expressed some subunits, suggesting that dopaminergic neurons are not the only cell type in substantia nigra capable of assembling the NADPH oxidase enzyme. Non-TH immunoreactive cell candidates that express NADPH oxidase may include other neuronal cell types, or more likely microglia and astrocytes [48], which when activated are known to express high levels of this enzyme [11,49].

**Figure 2:**
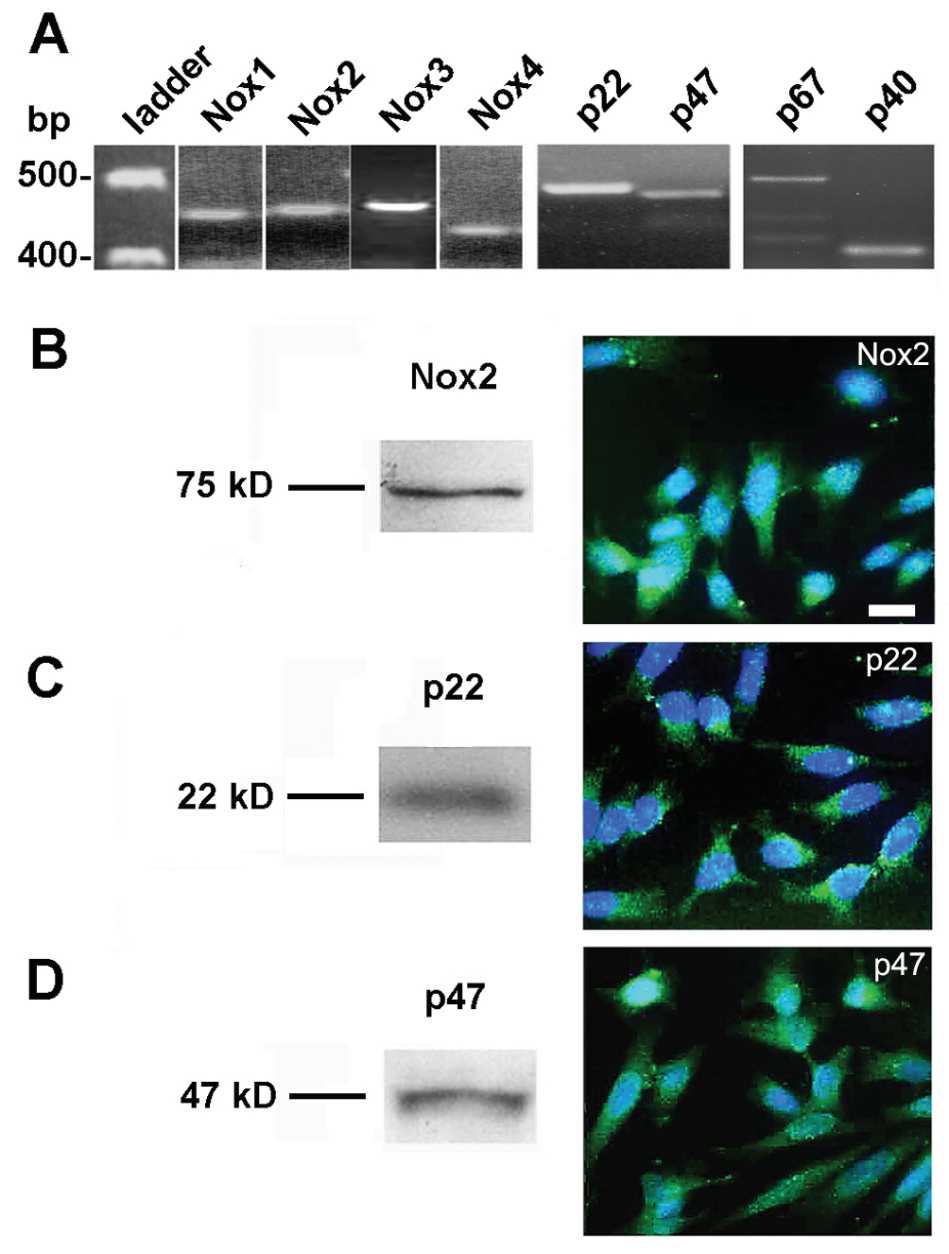
**Dopaminergic cells express subunits of the NADPH oxidase complex**. (A) mRNA from untreated N27 cells was reverse transcribed and amplified using PCR primers specific to rat NADPH oxidase subunits. Nox1-4 subunits as well as p22^phox^, p40^phox^, p47^phox^, and p67^phox ^were identified by their mRNA expression. Nox2, p22^phox^, and p47^phox ^were also detected by their protein expression (Western immunoblot, B-D) and cellular localization (immunofluorescence, B-D). Scale bar equals 10 μm; all three micrographs were taken at the same magnification.

Expression of mRNA for all the subunits of NADPH oxidase, including NADPH oxidase subunits p22^phox^, p47^phox^, and p67^phox^, as well as the cytosolic regulatory NADPH oxidase subunit p40^phox ^mRNA, which is less involved in superoxide production, and the four Nox homologues is present in the nigral dopaminergic neuronal cell line N27 (Figure 2A). Western blotting and immunofluorescence histochemistry of Nox2, p22^phox^, and p47^phox ^confirmed the translation of these mRNAs in N27 (Figure 2B-D). The presence of p67^phox ^was confirmed by fluorescence immunoreactivity in nigral dopaminergic neurons in Figure 1.

### The dopaminergic neurotoxin MPP+ induces an increase in intracellular ROS in the N27 dopaminergic cell line

**Figure 3:**
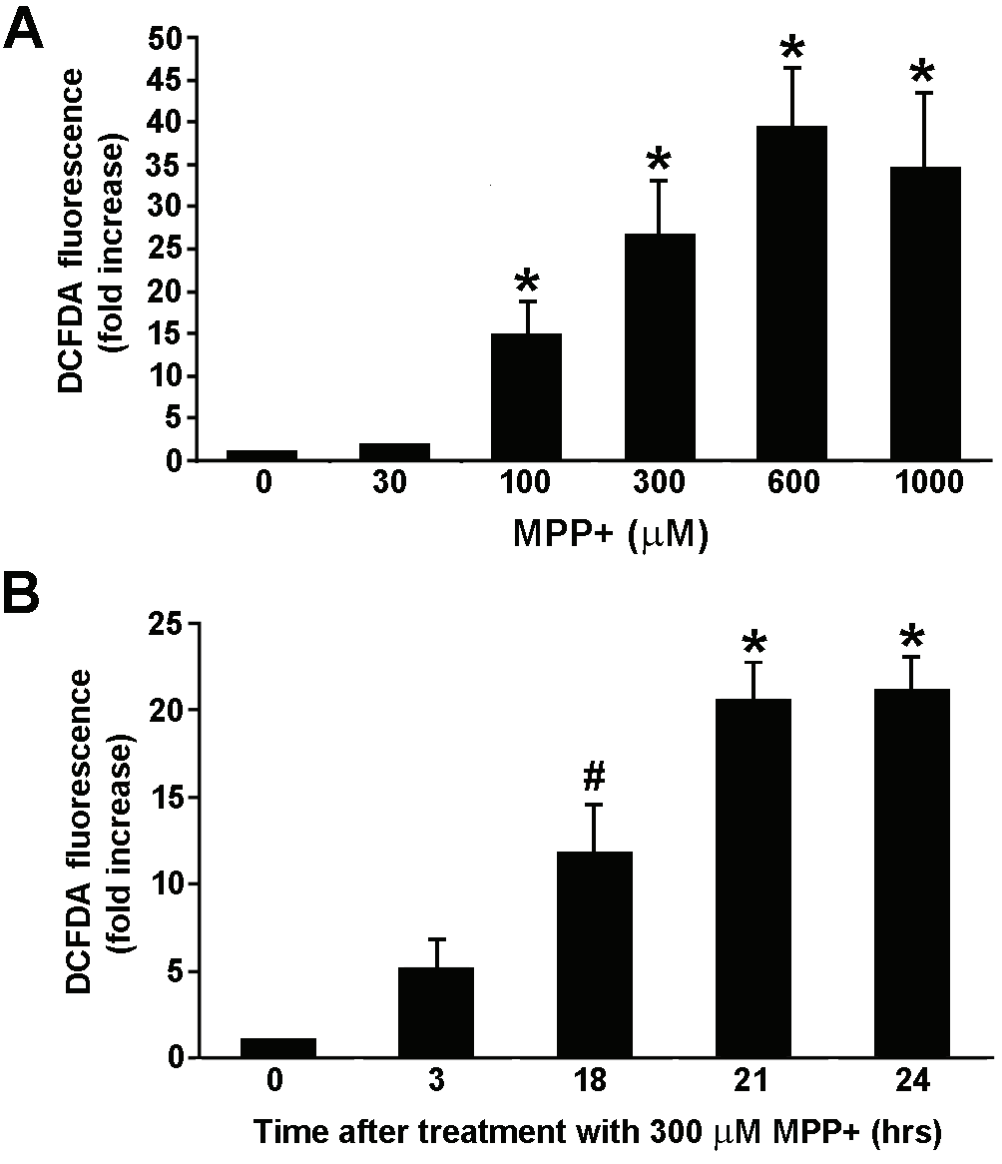
**MPP+ induces a dose- and time-dependent increase in intracellular ROS in the N27 dopaminergic cells**. (A) N27 cells were treated with increasing concentrations of MPP+ (up to 1000 μM) for 18 hours and H_2_O_2 _(ROS) levels were measured using carboxy-H_2_-DCFDA fluorescence and flow cytometry. ROS levels are reported as fold increase above values observed in cells that received no MPP+, control. * p < 0.01 compared to 0 μM MPP+ control. (B) N27 cells were treated with 300 μM MPP+ and ROS levels were detected at different times after treatment. # represents p < 0.05 compared to 0 hour and * represents p < 0.01 compared to 0 hour. Data are from 3 independent experiments with n = 6 wells per experiment.

MPP+ treatment of the dopaminergic N27 cell line served here as a surrogate *in vitro *model of the *in vivo *MPTP-treatment model of PD. In accordance with neurons in normal substantia nigra (Figure 1), N27 dopaminergic neurons have all the subunits necessary to produce superoxide via Nox pathways. Treating N27 cells with increasing amounts of MPP+, the active metabolite of MPTP, corresponded to a dose-dependent increase in the production of superoxide, which peaked at a 20-fold increase (Figure 3A). In addition, exposure to MPP+ at a constant level (300 μM) led to a time-dependent increase in H_2_O_2 _accumulation that was first detected at three hours and plateaued at 21 hours (Figure 3B).

### MPP+ binding to mitochondrial complex I engenders the initial wave of ROS production

**Figure 4:**
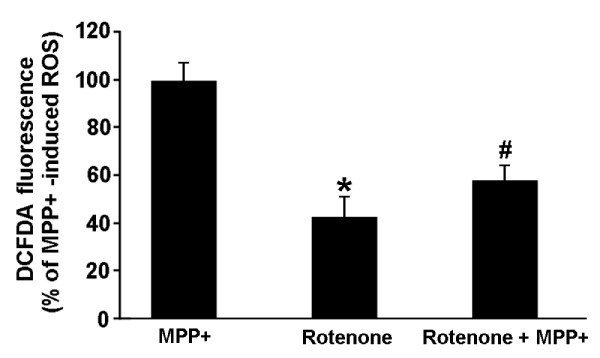
**Complex I inhibitor rotenone attenuates MPP+ induced ROS**. N27 cells were treated with MPP+ or rotenone alone, or combined for 18 hours. H_2_O_2 _(ROS) levels were measured at that time using carboxy-H_2_-DCFDA and flow cytometry and are reported as % of MPP+ induced ROS. * represents p < 0.01 and # represents p < 0.05, both compared to the MPP+ treated cells. Data are from 3 independent experiments with n = 6 wells per experiment.

The potential of MPP^+ ^binding to mitochondrial complex I in generation of ROS was previously shown in competition studies in which C^14^-labeled rotenone competed with MPP+ for binding to sub-mitochondrial particles [50]. Rotenone-treatment of MPP+ treated N27 cell cultures results in a fifty percent suppression of H_2_O_2 _production (Figure 4). As complex I is necessary for electron transport-dependent ATP production, it may be that MPP+ binding to mitochondrial complex I contributes to cell killing by suppressing ATP generation while increasing superoxide production.

### NADPH oxidase contributes to MPP+ induced ROS

**Figure 5:**
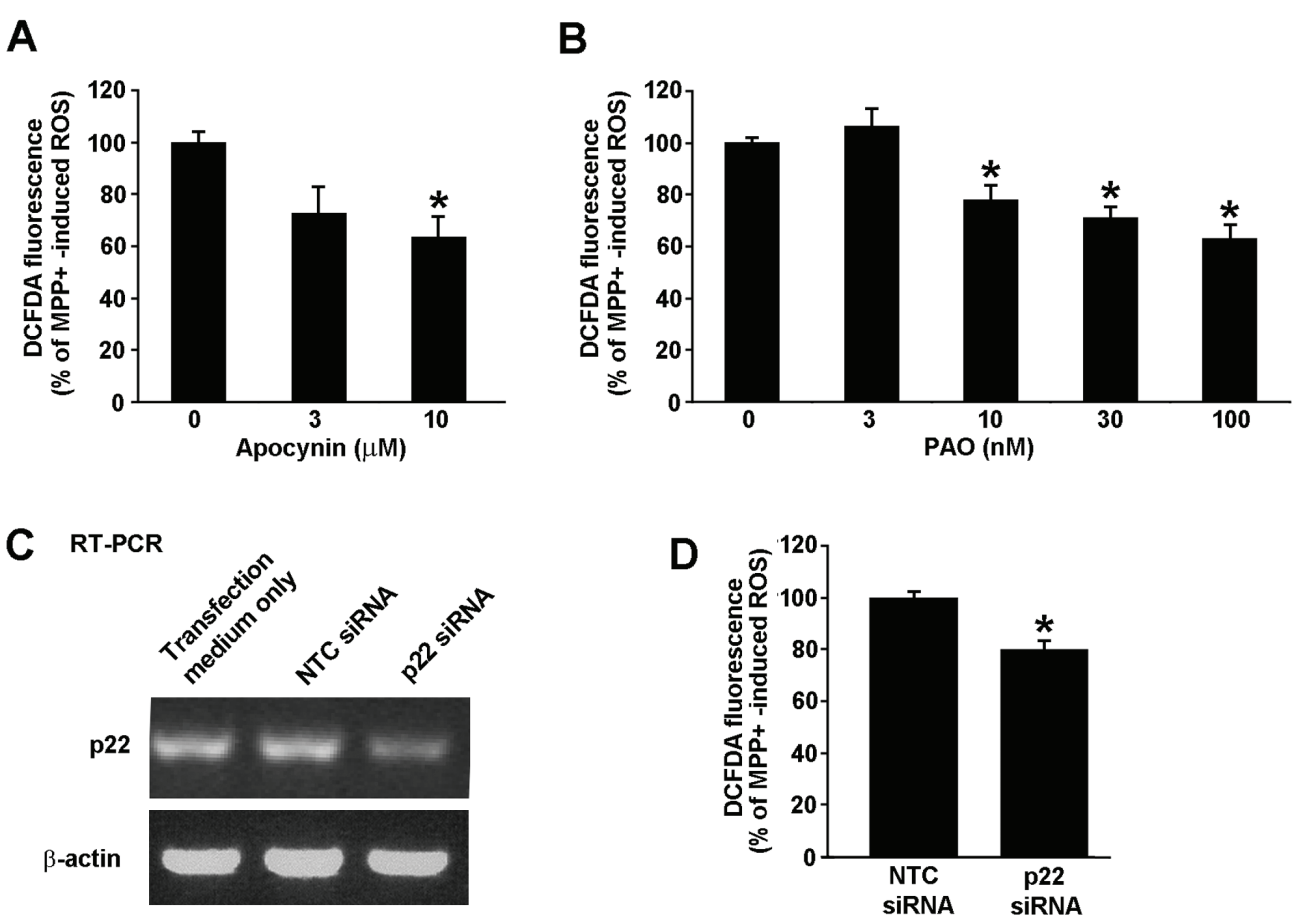
**Pharmacological inhibitors of NADPH oxidase and silencing p22**^**phox **^**using siRNA attenuate MPP+ induced ROS**. N27 cells were treated for 18 hours with 300 μM MPP+ and increasing concentrations of either apocynin (A) or phenylarsine oxide (PAO) (B). H_2_O_2 _levels were measured using caboxy-H_2_-DCFDA and flow cytometry. ROS levels are represented as percent of MPP+ induced ROS. * represents p < 0.05 compared to cells receiving no inhibitor. (C) N27 cells were transfected with a non-targeting control (NTC) siRNA or a SmartPool siRNA targeting p22^phox^. Total RNA was collected and reverse transcribed to cDNA. Primers complimentary to rat p22^phox ^were used to amplify the cDNA. An image of a single representative ethidium bromide-stained agarose gel is shown from one knockdown experiment out of three that produced an average knockdown of 40%. (D) N27 cells were transfected with NTC or p22^phox ^siRNA Smartpool and the intracellular H_2_O_2 _was measured with flow cytometry as described above. * represents p < 0.05 compared to NTC siRNA-treated cells. Data are from 3 independent experiments with n = 6 wells per experiment.

The role of NADPH oxidase in the response of N27 cells to MPP+ treatment was examined by treatment of cells for 18 hours with 300 μM MPP+ in the absence or presence of increasing concentrations of NADPH oxidase inhibitors phenylarsine oxide (PAO) or apocynin. Both NADPH oxidase inhibitors led to a reduction in NADPH oxidase activity in a dose dependent manner, with a maximum of 40% attenuation of the MPP+ induced NADPH oxidase-mediated ROS effect at doses of 10 μM apocynin (Figure 5A) and 100 nM PAO (Figure 5B). To further validate the role of the NADPH oxidase in MPP+ driven generation of ROS, we genetically suppressed the expression of the p22^phox ^subunit, which is necessary for the activity of all the Nox isoforms of NADPH oxidase. Such silencing of p22^phox ^mRNA resulted in a 40 percent reduction in p22^phox ^expression compared to a non-targeting control (NTC) siRNA (Figure 5C). Although the amount of p22^phox ^silencing in these cells is restricted by the limited transfection efficiency, the examination of ROS levels in p22^phox ^versus NTC siRNA-transfected cells revealed a significant 20% attenuation of MPP+ induced ROS production (Figure 5D). This gene silencing approach yielded a decrease in ROS production that was similar to that achieved by maximally effective concentrations of NADPH oxidase inhibitors PAO and apocynin.

### NADPH oxidase produces a delayed ROS response following MPP+ treatment suggestive of a second wave response

**Figure 6:**
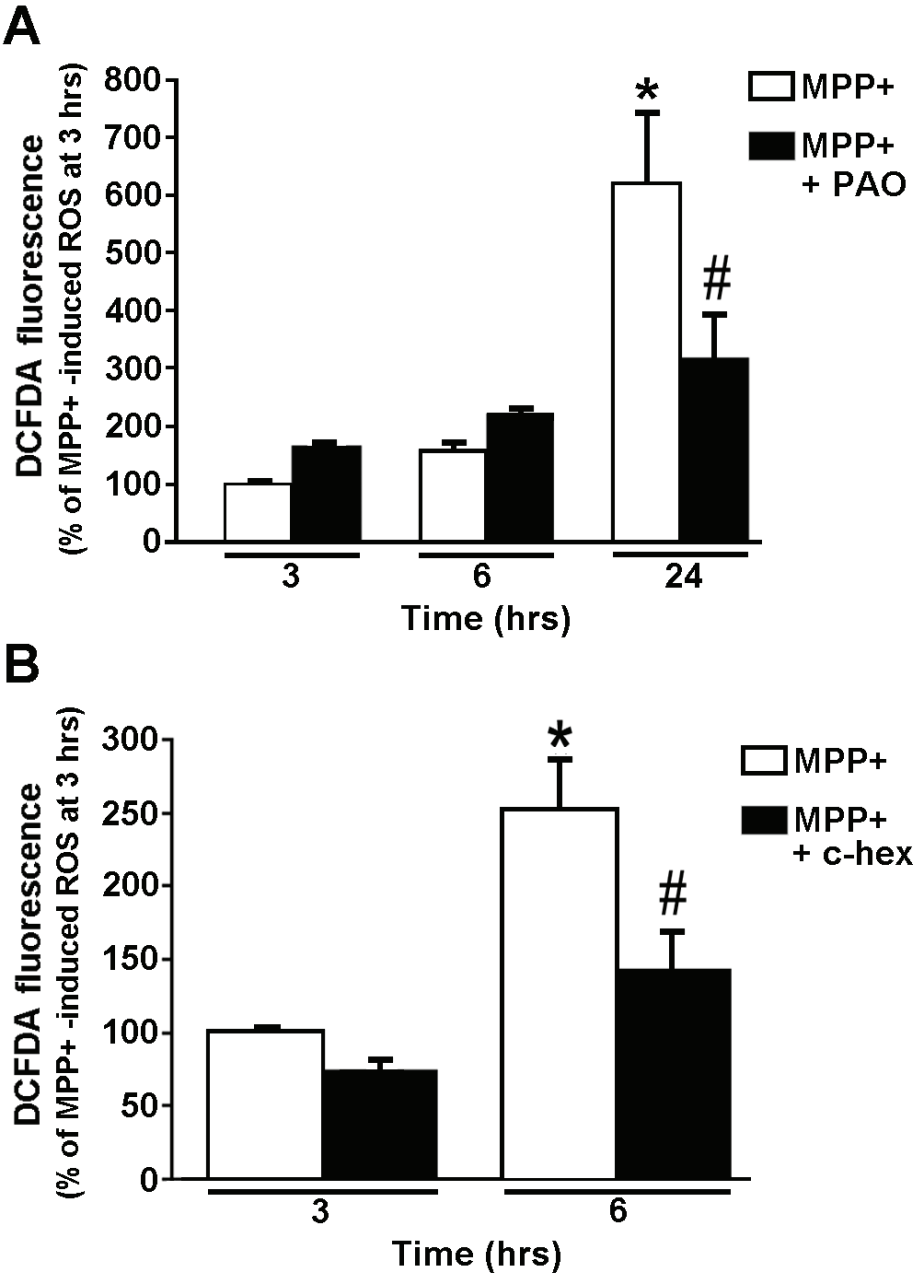
**NADPH oxidase inhibitors and an inhibitor of *de novo *protein synthesis, cyclohexamide, attenuate ROS, but only at later time points after initiation of the MPP+ treatment**. (A) N27 cells were treated with 300 μM MPP+ in the absence or presence of 10 nM PAO. Intracellular ROS levels were measured using carboxy-H_2_-DCFDA and flow cytometry at 3, 6, or 24 hours after initiation of the MPP+ treatment. * represents p < 0.001 compared to 3 hours MPP+ and # represents p < 0.001 compared to 24 hours MPP+. (B) N27 cells were treated with MPP+ for 3 or 6 hours in the absence or presence of the protein synthesis inhibitor cyclohexamide (c-hex). Intracellular H_2_O_2 _levels were measured as described above. Treatments are plotted as a percent of the 3-hour MPP+ treatment. * represents p < 0.001 compared to 3 hours MPP+ and # represents p < 0.05 compared to 6 hours MPP+. Data are from 3 independent experiments with n = 6 wells per experiment.

As MPP+ treatment led to an accumulation of ROS in a time-dependent manner (Figure 3B), N27 cell cultures were treated with MPP+ for three, 6, or 24 hours in the presence or absence of the NADPH oxidase inhibitor PAO. In the presence of PAO, increases in ROS levels were not suppressed by PAO until treatment times were prolonged to 24 hours, and this increase was not fully suppressed by PAO treatment (Figure 6A). This suggests that ROS production in response to MPP+ treatment occurs in two waves; the first detectable as early as 3 hours can be accounted for by MPP+ binding to mitochondrial complex I, a well known source of oxyradicals [51,52], followed, hours later, by the second wave arising from NADPH oxidase activation. The fact that NADPH oxidase inhibitors selectively suppressed ROS production is consistent with the idea that this second wave of ROS is mediated by extramitochondrial NADPH oxidase.

### *De novo *protein synthesis is required for the second wave of ROS: Potential role of Nox2 synthesis in ROS generation

As ROS are potent signaling molecules that regulate gene expression [53], we examined the possibility that ROS generation in MPP+ treated N27 cells requires protein synthesis. The presence of the protein synthesis inhibitor cyclohexamide had no effect on the MPP+ induced ROS levels after three-hours of inhibition, but treatment with cyclohexamide for 6 hours attenuated increase in ROS, suggesting that the second wave of ROS requires *de novo *synthesis of proteins (possibly NADPH oxidase subunits, Figure 6B).

**Figure 7:**
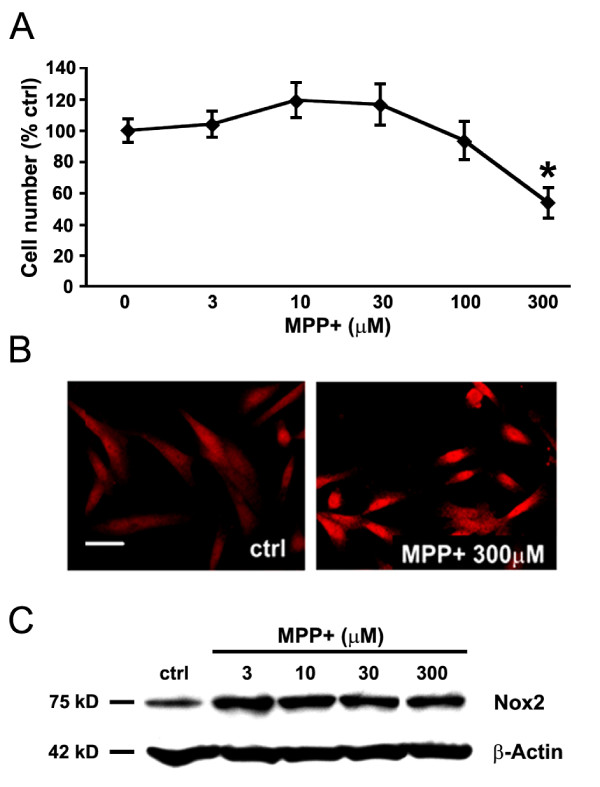
**Catalytic subunit of NADPH oxidase Nox2 is elevated by the MPP+ treatment**. N27 cells were treated with different concentrations of MPP+ for 24 hours. (A) Survival of N27 cells as a function of MPP+ concentration. (B) Immunofluorescent detection of Nox2 in N27 cells cultured either in the absence (control, ctrl) or presence of 300 μM MPP+, a concentration of MPP+ that results in loss of 45 percent of N27 cells. Scale bar equals to 20 μm. (C) Western immunoblot illustrating the effect of treating the cultures with increasing concentrations of MPP+ on expression of Nox2 protein. β-actin served as a loading control. Data in panel A, are from 3 independent experiments with n = 6 wells per experiment and *represents p < 0.01 compared to all other concentrations examined.

Treating N27 cells for 24 hours with 300 μM MPP+ resulted in death of 45 percent of these cells by that point in time (Figure 7A). This corresponded to an increase in Nox2 protein expression in these cells as determined by immunofluorescence (Figure 7B) and by Western immunoblotting (Figure 7C). Nox2 expression, measured by Western blot, was highly sensitive to MPP+ as it was increased even at MPP+ concentration of 3 μM, which was well below the 300 μM required for cell killing (Figure 7C).

### Angiotensin receptor blocker losartan suppresses MPP+ induced ROS generation

**Figure 8:**
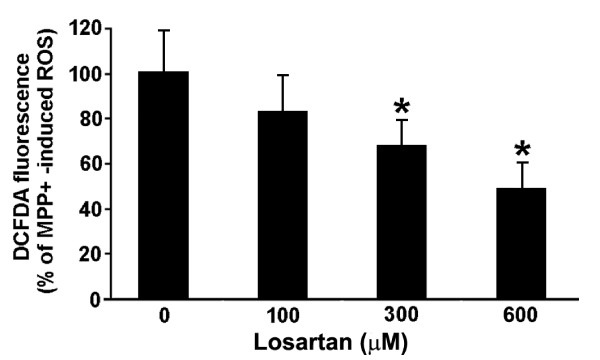
**Angiotensin receptor blocker losartan suppresses ROS production**. Co-treatment of N27 cultures for 18 hours with 300 μM MPP+ and with increasing concentrations of losartan results in dose-dependent reduction in MPP+ induced H_2_O_2 _production. Data are from 3 independent experiments with n = 6 wells per experiment and * represents p < 0.05 compared to culture receiving no losartan.

Based on our earlier finding that losartan, an angiotensin-receptor blocker, rescues nigral dopaminergic neurons in the MPTP mouse model of PD [54] via inhibition of the Nox pathway for superoxide generation [11], MPP+ treated N27 cultures were co-treated for 18 hours with increasing concentrations of losartan. Concentrations of losartan at both 300 and 600 μM reduced ROS generation by 30 and 50 percent, respectively (Figure 8).

## Discussion

Previously emphasis was on microglia as agents of dopaminergic neuron cell death in Parkinson's disease. This was based on findings of microglial cell involvement in 6-hydroxydopamine (6-OHDA)-induced superoxide production [55] and diminished mitochondrial ATP production in rat mesencephalic neuron/glia cultures [55,56]. However, our discovery of mechanisms by which dopaminergic neurons themselves may contribute to superoxide production adds a further dimension to our understanding of the ways in which such cell death occurs in the face of either environmental neurotoxin-induced or idiopathic PD. Furthermore, our demonstration that three of the NADPH oxidase subunits, Nox2, p47^phox^, and p67^phox ^are present in dopaminergic neurons in substantia nigra adds credence to a neuron cell autonomous contribution to the loss of nigral neurons in PD; a contribution that is over and above the known role of Nox2, p47^phox^, and p67^phox ^in microglial production of superoxide-induced cell death.

Here we provide evidence that ROS generation by dopaminergic neurons in response to MPP+ induced neurotoxic stress occurs in two distinct waves. The first wave is the result of MPP+ binding to mitochondrial complex I. The second wave requires protein synthesis for production of extra mitochondrial NADPH oxidase and ROS generation. Identification and characterization of this two-wave cascade of ROS generation provide insight into mechanistic intricacies involved in neurotoxin-stimulated N27 cell death. By analogy to the degeneration of dopaminergic neurons observed in PD models [43,49,57,58] and potentially in idiopathic PD [59,60], our findings provide several novel therapeutic targets. The present report is the first evidence from either dopaminergic or other neuron cell types of a chemical stressor, in this case MPP+, inducing two distinct waves of ROS generation that are characterized by both temporal and cellular compartment separation. The occurrence of an initial wave of ROS production, shown by rotenone competition with MPP+ for mitochondrial complex I ROS generation, which is followed hours later by a second wave of NADPH oxidase-generated ROS suggests that the total burden of a cell's ROS generation may be greater than the sum of wave one and wave two.

During the completion of the present study, there was a report of such a two wave response in serum starved human embryonic kidney cells [293 HEK(T)] [61]. Serum withdrawal in these cells led to initial elevation of ROS in the mitochondria, followed by generation of Nox-mediated ROS 4-8 hours later, an event dependent on Lyn tyrosine kinase. Silencing Nox1 attenuated this second wave of ROS production in 293 HEK(T) cells, an effect comparable to that observed here with silencing p22^phox ^in N27 cells. The notion that NADPH oxidase Nox2-related generation of ROS in neurons is largely extramitochondrial does not preclude generation of ROS from a second intramitochondrial source, as has been reported for Nox4 in mitochondria of cardiac myocytes [62] and of kidney cortical cells [63].

Demonstration of synthesis of Nox2, p47^phox ^and p67^phox^, NADPH oxidase subunits that are necessary for mitochondrial complex I-Nox responses to cellular stress in dopaminergic neurons in intact adult substantia nigra, together with evidence of a stress-elicited complex I-Nox response in a MPP+ treated dopaminergic cell line (N27) lends credence to the idea that these events are important in PD neuropathogenesis. For example, during hypoxia and reoxygenation of hippocampal and cortical neurons, NADPH oxidase plays a significant role in ROS accumulation at late stages of the stress response [64]. In breast and ovarian tumors, crosstalk between mitochondria and NADPH oxidase requires mitochondrial production of ROS and Nox1 [65], and loss of Nox1 signaling contributes to breast and ovarian tumorigenesis [65]. Because in the current study, the NADPH oxidase inhibitors fail to reduce ROS at early time points following MPP+, and only do so at later times, the reduction in ROS most likely occurred via inhibition of a cellular signaling pathway, and not because of any unforeseen ROS scavenging properties of the inhibitors themselves.

Preliminarily we showed that systemic treatment of mice with losartan, an angiotensin II receptor type 1 antagonist commonly prescribed anti-hypertensive, suppresses MPTP-induced dopaminergic neuron loss and dysfunction *in vivo *and *in vitro *[54]. However, the specific mechanisms whereby losartan's salutary effect is brought about was unknown until our current finding that treatment of dopaminergic cells with losartan attenuates MPP+ induced ROS with kinetics suggestive of a temporally-regulated, two-wave response. In addition, a characteristic distinguishing between the waves was shown by the observation that protein synthesis is required for superoxide production in the context of the second wave, but not the first. Thus, we can now say that MPP+ treatment of dopaminergic neurons elicits protein synthesis as a requirement for generation of NADPH oxidase subunit(s), including the catalytic subunit Nox2. Experiments using another neurotoxin, 6-OHDA, support such occurrence of *de novo *synthesis of NADPH oxidase subunits as a part of ROS generation in rat striatal and ventral midbrain tissues [55].

Although it is clear that superoxide is a potent signaling molecule that activates a multitude of signaling pathways [53], the mechanism by which oxidative stress and mitochondrial dysfunction leads to changes in gene expression is unknown. We show here for the first time that the stress-induced initial wave of ROS production comes from mitochondrial respiration, leads to the activation of signaling pathways involved in a second wave of ROS production that depends on protein generation required for assembly and phosphorylation of NADPH oxidase subunits. Therefore, it seems logical that the generation and phosphorylation of a cytosolic subunit(s) of NADPH oxidase is required for setting in motion events giving rise to the second wave. One such example has been suggested to be important in protein kinase C-mediated phosphorylation of p47^phox^, which is required for p47^phox ^translocation from the cytosol to the plasma membrane for the activation of the Nox2 subunit of the NADPH oxidase [14] and initiation of the second wave.

Although the functional significance of the second wave remains to be characterized in full, the two waves in the neuron resulting from—mitochondrial complex I inhibition and extramitochondrial NADPH oxidase activation —may play a role in preconditioning as an adaptive stress response (a.k.a. hormesis) in which brief exposure to a sub-lethal stressor fortifies cellular defenses in an effort to protect cells from a subsequent exposure to severe stress. Such hormetic effects could be explained, for example, by activation of AT1 receptor/Nox pathway by angiotensin II, which elevates activity of key antioxidant enzymes such as catalase, superoxide dismutase and glutathione peroxidase in rat hypothalamus [66]. Furthermore, mitochondrially produced ROS and ATP-sensitive potassium channels have been shown to play a role in the preconditioning machinery [67]. Whether neuronal ROS originating from the two waves act to balance such adaptive machinery awaits assessment.

**Figure 9:**
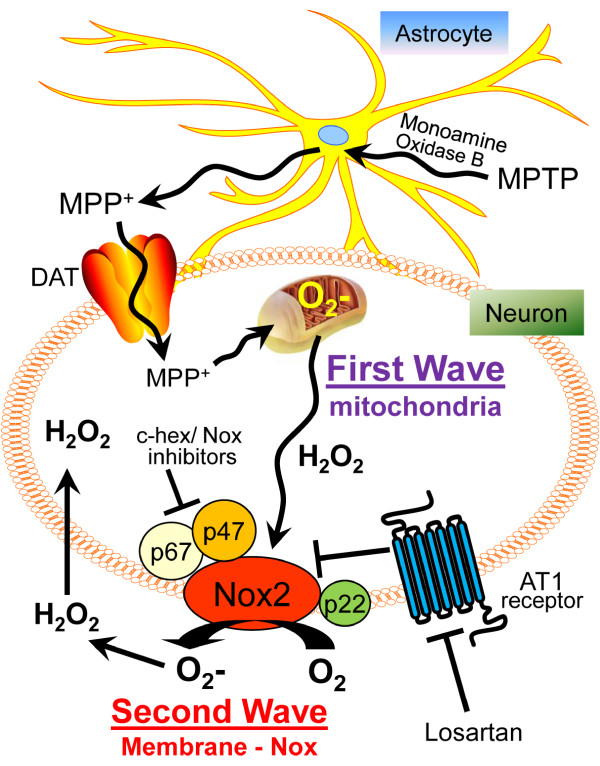
**A model of MPP+ induced generation of two waves of ROS in dopaminergic neurons and mechanisms of blockade by losartan**. MPTP is converted to MPP+ in astrocytes by monoamine oxidase B [45]. Specific uptake of MPP+ released from astrocytes into dopaminergic neurons occurs via a cell membrane dopamine transporter (DAT). After entering the cytosol, MPP+ binds and inhibits mitochondrial complex I, leading to an increase in mitochondrial ROS (First Wave), which, in turn, leads to the activation of the extramitochondrial NADPH oxidase complex to generate superoxide (Second Wave). The Second Wave of ROS can be blocked by pharmacological and genetic inhibition of NADPH oxidase, as well as inhibition of protein synthesis by cyclohexamide (c-hex), and by losartan blockade of AT1 receptor. Cell surface Nox2-generated superoxide is readily dismutated to H_2_O_2_, which can act either extracellularily or cross into the cytosol of dopaminergic neurons for propagation of the two-wave cascade. Reaction of superoxide with nitric oxide can generate highly cytotoxic peroxynitrite, which has been reported for its neurotoxicity in models of PD [74,75].

ROS generation is promoted by angiotensin II binding to the AT1 receptor, which induces a protein kinase C-Nox signaling cascade and leads to elaboration of superoxide from NADPH oxidase [19]. Losartan competes for binding to the AT1 receptor for suppression of angiotensin II-induced increases in ROS production (Figure 9). Although the existence of an extramitochondrial second wave is clear from our data, the specific mechanism(s) by which the mitochondria detect oxidative stress in dopaminergic cells and induce the second wave is unknown, one candidate for mitochondrial oxidative stress recognition has been proposed, *viz*., inactivation of mitochondrial aconitase, an iron-sulfur-containing enzyme necessary for ATP production. Increases in the release of ferrous iron from mitochondrial aconitase catalytic center suggest that iron may function as an oxidative stress biosensor [68,69]. However, it is conceivable that the Nox-induced ROS signals not only affect intracellular signaling pathways that precipitate the two-wave cascade of ROS generation and in this way may influence neighboring cells, including neurons, astrocytes, and microglia, all of which, as we show here in dopaminergic neurons, express NADPH oxidase [8,70]. In fact, CD200 ligand expressed on the surface of neurons, but not microglia, interacts with microglial CD200 receptor (CD200R) purportedly maintaining microglia in a resting state [71]. Reduced CD200/CD200R interactions between neurons and microglia may contribute to Parkinson [72] and Alzheimer pathogenesis [73] via activation of microglial NADPH oxidase.

## Conclusions

From our findings that NADPH oxidase subunits are universally expressed in nigral dopaminergic neurons in rats and mice, we conclude that these subunits contribute to ROS generation. The fact that rat N27 cells undergoing neurotoxic stress display a two-wave cascade of oxidative stress, as we show here by treating these cells with MPP+, is consistent with wave one being the result of the binding of MPP+ to mitochondrial complex I. Furthermore, the finding that the second wave can be suppressed by treatment with pharmacological inhibitors of NADPH oxidase implies that the second wave is the result of the activation of extra-mitochondrial NADPH oxidase. The existence of the two waves allows for segregation of ROS production into distinct sub-cellular compartments (Figure 9), suggesting that temporal and translational controls are critical for the trans-compartmental ROS signaling in neurons. Understanding of this process takes a key step toward development of more efficacious preventive or disease-modifying strategies for PD. In addition, such strategies may be useful in other neurodegenerative conditions that are aided and abetted by excessive ROS.

## List of Abbreviations

6-OHDA: 6-hydroxydopamine; AT1: angiotensin II type 1 receptor; ATP: adenosine 5'-triphosphate; CD: cell determinant; C-HEX: cyclohexamide; DAT: dopamine transporter; DCFDA: dichloro-fluorescein diacetate; HEK: human embryonic kidney; MPP+: 1-methyl-4-phenylpyridinium; MPTP: 1-methyl-4-phenyl-1,2,3,6-tetrahydropyridine; NADPH: nicotinamide adenine dinucleotide phosphate; NTC: non-targeting control; PAO: phenylarsine oxide; PD: Parkinson's disease; PHOX: phagocytic oxidase; ROS: reactive oxygen species; RT-PCR: reverse transcriptase polymerase chain reaction; TH: tyrosine hydroxylase.

## Competing interests

The authors declare that they have no competing interests.

## Authors' contributions

WMZ and MD developed the hypothesis, designed the experiments, and together with WSTG contributed to data analysis and writing of the manuscript. GPB performed experiments with inhibitors and flow cytometry and contributed to writing, JT, BM, DC, ALR conducted experiments and SMJ participated in experiments, data analysis and writing. All authors read and approved the final manuscript.
